# RETRACTION: Catechins and Sialic Acid Attenuate *Helicobacter pylori*–Triggered Epithelial Caspase‐1 Activity and Eradicate *Helicobacter pylori* Infection

**DOI:** 10.1155/ecam/9862067

**Published:** 2026-03-23

**Authors:** Evidence-Based Complementary and Alternative Medicine

RETRACTION: J. C. Yang, H. C. Yang, C. T. Shun, T. H. Wang, C. T. Chien, and J. Y. Kao, “Catechins and Sialic Acid Attenuate *Helicobacter pylori*–Triggered Epithelial Caspase‐1 Activity and Eradicate *Helicobacter pylori* Infection,” *Evidence-Based Complementary and Alternative Medicine* 2013, no. 1 (2013): 248585, https://doi.org/10.1155/2013/248585.

The above article, published online on 11 April 2013 in Wiley Online Library (https://wileyonlinelibrary.com), has been retracted by John Wiley & Sons Ltd. The retraction has been agreed due to multiple inconsistencies in the figures, initially identified on PubPeer [1]. Further investigation identified additional concerns. A summary of all figure concerns is as follows:

• In Figure 6, panels c3 and d3 appear to be duplicated
• In Figure 6, panels b2 and d2 appear to be duplicated
• Figure 5c and Figures 6 (c3 and d3) appear to be duplicated

The authors did not respond when notified of the retraction.
